# Predicted Chemotherapy Benefit for Breast Cancer Patients With Germline Pathogenic Variants in Cancer Susceptibility Genes

**DOI:** 10.1093/jncics/pkaa083

**Published:** 2020-09-18

**Authors:** Allison W Kurian, Kevin C Ward, Paul Abrahamse, Ann S Hamilton, Steven J Katz

**Affiliations:** 1 Department of Medicine and of Epidemiology and Population Health, Stanford University, Stanford, CA, USA; 2 Department of Epidemiology, Rollins School of Public Health, Emory University, Atlanta, GA, USA; 3 Department of Health Management and Policy, School of Public Health, University of Michigan, Ann Arbor, MI, USA; 4 Department of Internal Medicine, University of Michigan, Ann Arbor, MI, USA; 5 Department of Preventive Medicine, Keck School of Medicine, University of Southern California, Los Angeles, CA, USA

## Abstract

Breast cancer patients increasingly undergo genetic testing. To examine chemotherapy indications for germline pathogenic variant (PV) carriers, we linked results of germline testing to Georgia and California Surveillance, Epidemiology, and End Results registry records, including 21-gene recurrence score (RS) results, for breast cancer patients diagnosed in 2013-2017. All statistical tests were 2-sided. Patients (N=37 349) had RS results of whom 714 had *BRCA1*, *BRCA2*, *CHEK2*, *ATM*, *PALB2*, or Lynch syndrome (*MLH1*, *MSH2*, *MSH6*, *PMS2*) PVs. For women aged 50 years or older at breast cancer diagnosis, RS often exceeded the chemotherapy benefit threshold (≥26) with *BRCA1* (71.7% vs 14.4% with none; *P* <.001), *PALB2* (37.1%; *P* = .001), and *BRCA2* (44.3%; *P* < .001) PVs. Results were similar for women diagnosed at younger than 50 years of age. PVs in *BRCA1*, but not *BRCA2*, *PALB2*, *ATM*, *CHEK2*, or Lynch syndrome genes, were associated with elevated RS on multivariable analysis (*P* < .001). Results may inform RS testing decisions in breast cancer patients with PVs.

##  

Germline multiple-gene sequencing is increasingly common, and some guidelines endorse testing every breast cancer patient (1,2). Guidelines do not advise using germline results in treatment decisions for early stage breast cancer (3). However, a recent study suggests that some clinicians modify treatment based on germline results (4). This emphasizes the need for better understanding of chemotherapy’s benefit for patients with germline pathogenic variants (PVs).

Tumor gene expression profiling characterizes breast cancer prognosis and chemotherapy response, as demonstrated by a randomized trial of the 21-gene recurrence score (RS) in early stage estrogen receptor and/or progesterone receptor (ER/PR)–positive, HER2-negative disease (5). We sought to determine chemotherapy indications among PV carriers by linking the results of germline and RS testing to records of female breast cancer patients reported to the statewide, population-based Surveillance, Epidemiology and End Results (SEER) registries of Georgia and California.

We previously published details of the Georgia–California SEER Genetic Testing Linkage Initiative (1,4,6). Briefly, all women diagnosed with breast cancer at age 20 years or older from 2014 to 2017 and reported to the California and Georgia registries by August 30, 2019, were linked with results of germline testing from 4 participating laboratories (Ambry Genetics, Aliso Viejo, CA; GeneDx, Gaithersburg, MD; Invitae, San Francisco, CA; Myriad Genetics, Salt Lake City, UT). Laboratories reported results as provided to the ordering clinician: pathogenic or likely pathogenic (combined for analysis as PV), variant of uncertain significance, and benign or likely benign (combined for analysis as negative) but omitted specific sequence changes because of privacy concerns. SEER provided all other variables including RS, which was also obtained by linking SEER data with the testing laboratory (Exact Sciences, Redwood City, CA) (7).

Patients were included if eligible for RS testing, defined as having stage I-II tumors (T stage 1-2, N stage 0-1) that were ER/PR-positive and HER2-negative (8). We limited the analysis of germline testing to breast cancer–associated genes in which PVs were detected frequently enough to provide sufficient cases for analysis (n ≥ 20): *ATM*, *BRCA1*/2, *CHEK2*, and *PALB2*. We included the Lynch syndrome (LS)–associated genes as a single category (*MLH1*, *MSH2*, *MSH6*, and *PMS2*), because they are commonly tested on multiple-gene panels, but their breast cancer association is uncertain (1,3). Women with PVs in other genes were excluded (n = 459).

Two-sided *P* values were calculated using 1-way Analysis of Variance (ANOVA) tests. Multivariable modeling was used to adjust for potential confounders of the association between germline results and RS, including race and ethnicity, age, tumor size, grade, and lymph node involvement. We examined the proportions of patients aged younger than 50 years with RS of 16 or higher and aged 50 years or older with RS of 26 or higher, the age-specific thresholds for chemotherapy benefit in the TAILORx trial, according to germline results (5). All statistical tests were 2-sided, and a *P* value of less than .05 was considered statistically significant.

A total of 199 201 women were diagnosed with breast cancer in California and Georgia during 2014-2017. Among these, 108 058 were RS eligible of whom 37 349 (34.5%) had RS results; of these, 11 257 (30.1%) were also linked to germline testing results. A total of 714 women had a PV in *ATM*, *BRCA1/2*, *CHEK2*, *PALB2*, or LS genes.

Among women diagnosed at age 50 years or older, mean RS was highest among *BRCA1* PV carriers (mean RS = 26.7, 95% confidence interval [CI] = 21.8 to 31.6), followed by *PALB2* (mean RS = 24.8, 95% CI = 21.3 to 28.4) and *BRCA2* PV carriers (mean RS = 23.3, 95% CI = 21.4 to 25.2), with each having higher mean RS than patients with negative (mean RS = 16.8, 95% CI = 16.7 to 17.1), variant of uncertain significance (mean RS = 17.2, 95% CI = 16.7 to 17.8), or no germline results (mean RS = 16.8, 95% CI = 16.7 to 16.9; Table 1). The association between RS and germline results did not vary by age. On multivariable modeling, PVs in *BRCA1* (*P* < .001) were associated with higher RS. Tumor grade was the most important confounder in the multivariable model, with grade 3 being statistically significantly associated with higher RS and with PVs in *BRCA1*, *BRCA2*, and *PALB2*. The proportion of patients with RS of 26 or higher followed a similar pattern: for carriers of PVs in *BRCA1*, it was 71.7%, in *PALB2* 37.1%, and in *BRCA2* 44.3% compared with 14.4% among patients testing negative (*P* ≤.001 for each comparison Figure 1). Results followed a similar pattern for women aged younger than 50 years with a threshold RS of 16 or higher (Table 1).

**Table 1.** Patient characteristics and 21-gene recurrence score according to germline genetic testing results

Germline testing results by age at cancer diagnosis^a^	No.^b^	% PV^c^	Race and ethnicity, %				T2, %	N1, %	Grade 3, %	High RS, %^d^	Mean RS (95% CI)	Mean RS adjusted (95% CI)^e^
			NHW	Black	Asian	Hispanic						
Age <50 y												
*BRCA1* PV	47	1.02	46.8	21.2	10.6	21.3	17.1	21.3	61.7	89.4	36.7 (31.8 to 41.7)	27.9 (22.2 to 33.5)
*BRCA2* PV	128	2.77	53.9	9.4	14.8	21.9	20.7	27.6	35.2	85.3	24.1 (22.3 to 25.9)	20.6 (15.4 to 25.9)
*PALB2* PV	23	0.72	60.9	4.4	21.7	13.0	16.7	8.7	43.5	73.9	23.1 (18.7 to 27.5)	17.7 (11.4 to 23.9)
*ATM* PV	27	0.88	70.4	7.4	7.4	14.8	12.0	22.2	25.9	62.9	18.3 (15.7 to 20.9)	15.4 (9.6 to 21.2)
*CHEK2* PV	69	2.24	89.3	1.9	1.9	6.8	13.4	19.4	18.4	56.5	17.9 (15.9 to 19.7)	14.9 (9.6 to 20.1)
Lynch PV^f^	22	0.42	59.1	4.6	22.7	13.6	17.7	18.2	27.3	36.4	14.5 (11.0 to 18.1)	10.7 (4.1 to 17.2)
VUS	834	—^g^	70.3	10.3	10.8	8.6	15.6	17.0	15.7	52.0	17.4 (16.8 to 18.1)	17.3 (16.7 to 17.9)
Negative	3475	—	80.3	6.6	5.4	7.7	18.9	17.9	14.7	52.5	17.5 (17.2 to 17.8)	17.2 (16.9 to 17.5)
No testing	2894	—	66.7	9.7	11.1	12.5	19.7	16.9	15.4	53.4	17.7 (17.3 to 18.0)	17.4 (17.0 to 17.7)
Age ≥50 y												
*BRCA1* PV	46	0.72	62.5	16.7	12.5	8.3	8.3	12.5	41.7	71.7	26.7 (21.8 to 31.6)	22.6 (16.9 to 28.2)
*BRCA2* PV	131	2.04	61.0	11.0	12.1	15.8	30.5	19.5	42.6	44.3	23.3 (21.4 to 25.2)	18.2 (12.9 to 23.4)
*PALB2* PV	35	0.76	89.5	5.3	0.0	5.3	21.1	5.3	26.3	37.1	24.8 (21.3 to 28.4)	21.1 (15.5 to 26.6)
*ATM* PV	54	1.20	77.8	14.8	3.7	3.7	37.0	18.5	25.0	20.4	20.4 (18.0 to 22.7)	18.2 (12.7 to 23.7)
*CHEK2* PV	103	2.28	86.5	3.9	1.9	7.7	25.0	19.2	19.2	16.4	18.8 (17.3 to 20.3)	17.0 (11.8 to 22.2)
Lynch PV^f^	29	0.11	81.8	0.0	0.0	18.2	18.2	18.2	27.3	24.1	19.7 (15.2 to 24.2)	15.8 (10.0 to 21.6)
VUS	1155	—	70.3	10.3	10.8	8.6	15.6	17.0	15.7	15.7	17.2 (16.7 to 17.8)	17.0 (16.4 to 17.5)
Negative	4859	—	80.3	6.6	5.4	7.7	18.9	17.9	14.7	14.4	16.8 (16.5 to 17.1)	16.8 (16.5 to 17.1)
No testing	23 106	—	66.7	9.7	11.1	12.5	19.7	16.9	15.3	14.7	16.8 (16.7 to 16.9)	16.7 (16.5 to 16.8)

a Genes are listed in order of descending 21-gene recurrence score. CI = confidence interval; NHW = non-Hispanic White; PV = pathogenic variant; RS = 21-gene recurrence score; T2 and N1 = American Joint Committee on Cancer staging variables from SEER registry; VUS = variant of uncertain significance.

b Excludes 68 patients with pathogenic variants in other genes.

c Proportion of patients tested for the gene who carried a PV.

d RS exceeding the threshold value for benefit of chemotherapy: RS ≥16 for younger than 50 years of age, RS ≥ 26 for 50 years and older.

e Margins were derived from a generalized linear model of RS, including race and ethnicity, tumor size, lymph node involvement, and grade as covariates.

f Lynch syndrome genes, analyzed collectively: *MLH1*, *MSH2*, *MSH6*, *PMS2*.^g^ not applicable.

**Figure 1.**
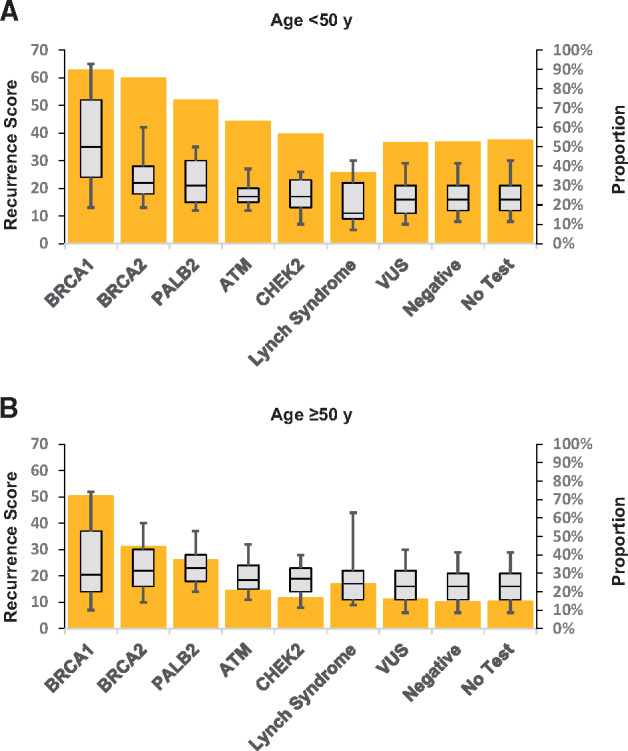
21-gene recurrence score by germline testing results and age at breast cancer diagnosis. A) 21-gene recurrence score (**box plots**) by germline testing result for women diagnosed at younger than 50 years of age and % with score ≥16 (columns). Boxes indicate 25th, 50th, and 75th percentiles of recurrence score (left scale). **Whiskers** indicate 9th and 91st percentiles. **Columns** indicate proportion with recurrence score ≥16 (right scale). Lynch syndrome genes, analyzed collectively: *MLH1*, *MSH2*, *MSH6*, *PMS2.* **B)** 21-gene recurrence score (**box plots**) by germline testing result for women diagnosed at age 50 years and older and % with score ≥26 (**columns**). **Boxes** indicate 25th, 50th, and 75th percentiles of recurrence score (left scale). **Whiskers** indicate 9th and 91st percentiles. **Columns** indicate proportion with recurrence score ≥26 (right scale). Lynch syndrome genes, analyzed collectively: *MLH1*, *MSH2*, *MSH6*, *PMS2*. VUS = variant of uncertain significance.

We integrated genetic testing results with data from the population-based SEER registries of Georgia and California to characterize predicted chemotherapy response among germline PV carriers. Our results are generally consistent with prior studies that reported higher mean RS among *BRCA1/2* PV carriers;

however, we found lower mean RS among *BRCA2* PV carriers than in prior studies. This difference may reflect the concentration of patients with worse tumor prognostic features among the tertiary referral samples used in prior studies, in contrast to our population-based sample (9-12). One previous study reported higher RS and PVs in *BRCA1/2*, but not in other breast cancer–associated genes combined; however, small numbers limited its statistical power (13). With more *ATM*, *CHEK2*, and *PALB2* PV carriers, we could analyze these genes separately and detected a trend toward higher RS among *PALB2* PV carriers, second in magnitude only to *BRCA1*. These results build on studies of *PALB2* epidemiology that suggest similar cancer spectrum and risk as with *BRCA2* PVs (14), perhaps reflecting similar tumor biology and chemotherapy response. The proportion of RS-eligible tumors varies substantially by affected gene, with more ER/PR-negative tumors among women with *BRCA1* and *PALB2* PVs. Our results suggest that breast cancers among carriers of PVs in *ATM* and *CHEK2*, which are relatively common and more typically ER/PR-positive, may behave similarly to those of women testing negative for germline PVs.

We found no difference by age in mean RS. However, the TAILORx trial demonstrated that the threshold RS value for chemotherapy benefit varies by age. For patients diagnosed at age younger than 50 years of age, a threshold RS value of 16 or higher should be considered (5,15). Our findings suggest that most RS-eligible *BRCA1/2* and *PALB2* PV carriers diagnosed with breast cancer at younger than 50 years of age (RS ≥16: *BRCA1 *=* *89.4%, *BRCA2 *=* *85.3%, *PALB2 = *73.4%), and many at age 50 years and older (RS ≥26: *BRCA1 *=* *71.7%, *BRCA2 *=* *44.3%, *PALB2 = *37.1%), have results indicating benefit from chemotherapy. However, it is important to note that the prognostic and predictive value of RS has not been studied among PV carriers, which is a key question for future research.

This study has limitations. There were few carriers of PVs in genes other than *ATM*, *BRCA1/2*, *CHEK2*, *PALB2*, and the LS genes. We have not yet evaluated cancer recurrence or mortality in association with germline or RS testing results. As in any clinically tested, real-world sample, there is the inherent bias that clinicians selected patients into testing. Although patients were from 2 large states, the cohort may not represent the entire US population. These limitations are balanced by considerable strengths: notably, this is the largest, most racially and ethnically diverse and population-based sample in which this question has been addressed, with detailed germline and RS results obtained from testing laboratories.

*BRCA1* PV carriers diagnosed with breast cancer at any age are statistically significantly more likely to have RS that indicates benefit from chemotherapy;

those with *PALB2* PVs also show a trend toward higher RS. These results may inform RS testing and chemotherapy decisions among breast cancer patients who carry cancer susceptibility gene PVs.

## Funding

Research reported in this publication was supported by the National Cancer Institute (NCI) of the National Institutes of Health under award number R01 CA225697 to Stanford University. The collection of cancer incidence data in Georgia was supported by contract HHSN261201800003I, Task Order HHSN26100001 from the NCI and cooperative agreement 5NU58DP006352-03–00 from the Centers for Disease Control and Prevention (CDC). The collection of cancer incidence data used in this study was supported by the California Department of Public Health pursuant to California Health and Safety Code Section 103885; CDC’s National Program of Cancer Registries, under cooperative agreement 5NU58DP006344; the NCI’s Surveillance, Epidemiology and End Results Program under contract HHSN261201800032I awarded to the University of California, San Francisco, contract HHSN261201800015I awarded to the University of Southern California, and contract HHSN261201800009I awarded to the Public Health Institute, Cancer Registry of Greater California. The ideas and opinions expressed herein are those of the author(s) and do not necessarily reflect the opinions of the State of California, Department of Public Health, the NCI, and the CDC or their contractors and subcontractors.

## Notes

**Role of the funders:** The funders had no role in the design and conduct of the study; collection, management, analysis, and interpretation of the data; preparation, review, or approval of the manuscript; and decision to submit the manuscript for publication.

**Disclosures:** Allison W. Kurian, MD, MSc, reports research funding to her institution for an unrelated project from Myriad Genetics. The other authors declare no conflicts of interest.

**Author contributions**: Allison Kurian: conceptualization, investigation, writing—original draft preparation, funding acquisition Kevin Ward: data curation, methodology, writing—review and editing, funding acquisition Paul Abrahamse: formal analysis, software, methodology, visualization, writing—review and editing Ann Hamilton: data curation, methodology, writing—review and editing, funding acquisition Steven Katz: conceptualization, investigation, writing—original draft preparation, funding acquisition.

**Previous presentation:** Preliminary results in partial form were submitted for presentation at the San Antonio Breast Cancer Symposium in San Antonio, TX, December 2019.

## Data Availability

Paul Abrahamse and Allison Kurian are independent of any commercial funder, had full access to all the data in the study and take responsibility for the integrity of the data and the accuracy of the data analysis.
